# Short-Term Heat Treatment of Ti6Al4V ELI as Implant Material

**DOI:** 10.3390/ma13214948

**Published:** 2020-11-04

**Authors:** Phuong Thao Mai, Therese Bormann, Robert Sonntag, Jan Philippe Kretzer, Jens Gibmeier

**Affiliations:** 1 Institute for Applied Materials, Karlsruhe Institute of Technology, 76131 Karlsruhe, Germany; phuong.mai@kit.edu; 2 Laboratory of Biomechanics and Implant Research, Clinic for Orthopedics and Trauma Surgery, Heidelberg University Hospital, 69118 Heidelberg, Germany; therese.bormann@med.uni-heidelberg.de (T.B.); robert.sonntag@med.uni-heidelberg.de (R.S.); philippe.kretzer@med.uni-heidelberg.de (J.P.K.)

**Keywords:** phase transformation, titanium alloy, short-term heat treatment, dilatometer

## Abstract

Due to its mechanical properties and good biocompatibility, Ti6Al4V ELI (extra low interstitials) is widely used in medical technology, especially as material for implants. The specific microstructures that are approved for this purpose are listed in the standard ISO 20160:2006. Inductive short-term heat treatment is suitable for the adjustment of near-surface component properties such as residual stress conditions. A systematic evaluation of the Ti6Al4V microstructures resulting from short-term heat treatment is presently missing. In order to assess the parameter field that leads to suitable microstructures for load-bearing implants, dilatometer experiments have been conducted. For this purpose, dilatometer experiments with heating rates up to 1000 °C/s, holding times between 0.5 and 30 s and cooling rates of 100 and 1000 °C/s were systematically examined in the present study. Temperatures up to 950 °C and a holding time of 0.5 s led to microstructures, which are approved for medical applications according to the standard ISO 20160:2006. Below 950 °C, longer holding times can also be selected.

## 1. Introduction

The average lifetime of an artificial hip joint in a patient can easily achieve 20 years. Due to demographic developments the number of revision operations is expected to greatly increase [1]. In order to facilitate these revisions, the majority of artificial hip joints are based on a modular construction involving taper junctions. However, micromotion occurring at the taper junction can lead to fretting corrosion and thus to wear at the joint surfaces, which may ultimately cause the implant to fail [2,3]. Furthermore, this failure pattern can trigger biological reactions in response to metallic debris. Possible consequences include toxicity, muscular atrophy, osteolysis and the development of pseudo tumors [4,5,6]. The design of the taper junction should therefore aim to minimize fretting corrosion. Additionally, preventing body fluids from entering the area of contact of the taper junction might lower the susceptibility of the joint materials for galvanic corrosion and increase the corrosion resistance of the taper junction.

In engineering terms, the taper junction between hip stem and artificial femoral head can be classified as a frictionally engaged shaft–hub connection [7]. The transfer of external forces and torques occurs through stiction, which in turn depends on the material pairing and the surface properties of the area of contact (e.g., surface roughness).

During conventional taper manufacturing, the taper surface is machined by turning of the taper with rather large feed rates, which results in a defined surface roughness [8]. Preliminary experiments on commercially available Ti6Al4V-ELI (extra low interstitials) tapers showed that the turning procedure induces compressive residual stress with an effective depth of up to about 100–150 μm in the near surface region. It is expected that these machining induced residual stresses will certainly affect the resulting stress state after joining of the parts. However, regarding the turning parameters applied, machining can induce either tensile or compressive residual stresses in the surface near region, which further depends on the wear of the cutting tool at the time of manufacturing. Furthermore, it is known that relatively rough machining can results in an alternating residual stress distribution following the surface roughness [9]. Due to the uncertain status quo that can affect the joint performance, we aim at erasing the prehistory of the parts processing and at the introduction of a well-defined residual stress distribution. Amongst others this can be realized through defined local thermal post treatment, where only the joining surfaces will be affected, e.g., by means of localized short-term heat treatment using induction heating. By this means, the near surface region only can be heat treated when using high frequencies due to the underlying skin effect [10]. Using an appropriate choice of the process parameters, as e.g., maximum temperature, frequency and cooling rate, the residual stress distributions can be adjusted to certain extend. The most significant effects can be expected at high temperatures and fast cooling rates, e.g., realized through self-quenching. From the heat conductance of titanium alloys, it can be expected that for self-quenching, maximum cooling rates of 1000 °C/s and more occur. However, the proper adjustment of process requires knowledge about the microstructure evolution during short-term heat treatments of Ti6Al4V. In particular for medical applications it must be assured that the microstructure of Ti6Al4V resulting from these inductive heat treatments adheres to the relevant standards [9,10]. In particular, a α-β-structure is required and thereby martensite structures are explicitly exempted. For this reason, the study aims to characterize the microstructural changes that occur during defined short-term heat treatments.

Unfortunately, there are no comprehensive, reliable data available concerning short-term heat treatments of Ti6Al4V alloys in the range of the β-transus-temperature. The few studies that have dealt with this topic lack data concerning cooling and quenching rates. First insights into material behavior in regard to short-term heat treatments can be drawn from the works of Elmer et al. [11], which relate to in situ synchrotron welding experiments on the alloy Ti6Al4V. The welding experiments showed that the heating rate and thus the transformation temperature decreased with increasing distance from the process zone. In another study, the authors took a closer look at the effect of different heating rates (2–30 °C/s) on the α-β transformation. The study indicated that inside the α-β field, increasing the heating rate led to a delay in the transformation from α to β.

The effect of the cooling rate on the developed microstructure when cooling down from the β-field was examined in [12]. The cooling rates used in dilatometry experiments ranged from 0.012 °C/s to 23.1 °C/s. For the rate of 23.1 °C/s, the dilatometry courses showed an increase in volume between 810 and 710 °C that can be attributed to a martensitic transformation. For low cooling rates (<2.5 °C/s), the beginning of the transformation was shifted to higher temperatures and only ended when reaching significant lower temperatures. At the start of the transformation, a sudden decrease in volume was observed. It is to be noted that no martensite could be detected after the experiment was concluded. The decrease in cooling rate had a significant impact on the structural morphology. An increase in lamellae and grainsize as well as a decrease in hardness could be observed as the cooling rate decreased. Ahmed et al. [13] studied cooling rates between 525 and 1.5 °C/s by means of a modified Jominy test. For the highest quenching rate of 525 °C/s they observed a complete martensitic transformation. For cooling rates between 410 and 20 °C/s they observed a “massive” martensitic transformation that was replaced by a transformation increasingly controlled by diffusion and the development of a Widmanstätten structure.

What all studies have in common is that relatively high cooling rates were considered and that the samples are annealed at temperatures above the β-transus-temperature (usually above 950 °C) before being quenched. Additionally, the samples are kept at these high temperatures long enough to become completely homogenous. Incomplete homogenization, which typically occurs during short-term heat treatments lasting seconds or even less, is not considered. As of now, the highest quenching rate that has been studied in this context is 525 °C/s. However, current studies suggest that the transformation behavior of the alloy Ti6Al4V would be permanently altered by higher quenching rates. The present studies indicate that higher quenching rates clearly result in a reduction of the martensite start temperature. A process where high cooling rates certainly affect the local microstructure evolution are additive manufacturing (AM) processes as, e.g., selected laser melting (SLM). Recent investigations on SLM for Ti6Al4V indicate, that the local reheating followed by a fast cooling down by self-quenching result in martensitic structures (e.g., [14]). To regain ductility, post-heat treatments or hot isostatic pressing (HIP) are carried out on the SLM-processed parts. At this, by HIP processing the martensitic needle-like α′ structure in the as-fabricated condition is transformed into the α + β structure (e.g., [15]). Hence, from systematic investigation on fast quenching of the alloy Ti6Al4V ELI, conclusions about the microstructure evolution during AM-processing can also be drawn.

In this first approach, we intend to fill that lack of knowledge regarding short-term heat treatments of the alloy Ti6Al4V ELI. The objective of the study is to characterize the transformation kinetics of Ti6Al4V ELI for very high heating and cooling rates by specific dilatometric short-term heat treatments on thin-walled hollow samples. Maximum temperatures are selected in the range between 650 and 1000 °C, i.e., the main focus of the investigations is on the temperature range below the beta transus temperature, which is at about 975 °C. In addition, different holding times of 0.5, 10 and 30 s during annealing at maximum temperature are chosen in order to obtain a closed database and to delimit the process window as to whether a short-term heat treatment is feasible in order to completely avoid martensite formation and to adjust a microstructure that is permitted for medical applications of Ti6Al4V ELI according to the standards [16] and [17].

## 2. Materials and Methods

The samples are made of the α-β Ti6Al4V ELI (medical grade) as defined in [18], which was provided as rod material with a diameter of 15 mm. The as received material was initially heat treated at 900 °C for 10 min under vacuum followed by oil quenching. Finally, the material was annealed at 500 °C for 4 h followed by a slow furnace cooling for stress relief and to provide a normalized microstructure for the following investigations. In Table 1, the same mechanical properties of the base material were listed as determined by means of tensile or microhardness testing, respectively. Hereby, the microhardness of the initial state was determined using a Vickers hardness tester type Q10 A+ from Qness, Golling an der Salzach, Austria using a load of 100 g. A total of 10 indentations were evaluated and an average value is given in Table 1.

**Figure 1:**
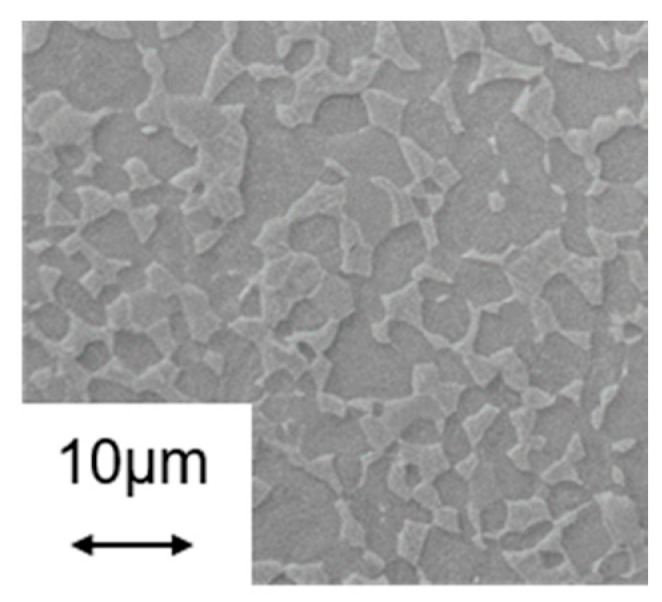
Scanning electron microscopy (SEM) image of the initial microstructure.

The resulting microstructure is a globular α-β structure with a β content of about 5.8% ± 0.6%. (determined by means of quantitative X-ray diffraction phase analysis) and an average grain size of about 4–5 μm (see scanning electron microscopy (SEM) image in Figure 1). EDS analysis (energy dispersive X-ray spectroscopy) showed that the elemental distribution over the materials volume is homogeneous and is in accordance with the nominal composition.

The samples for the dilatometer experiments are hollow cylinders with a wall thickness of 0.5 mm and an average length of 10 mm. The outer diameter is 4 mm. For temperature control, a thermocouple type S was spot welded in the middle of the sample length. A dilatometer type DIL 805 from TA Instruments (New Castle, DE, USA) was used for the studies. The sample was held between two push rods made of fused silica with a holding force of approx. 10 N. To avoid oxidation, the chamber was evacuated and flooded with helium and the complete short-term heat treatment was performed under helium inert gas atmosphere. The samples were heated with a heating rate of 1000 °C/s to the target temperatures 600, 750, 850, 950 and 1000 °C, respectively. When the target temperature was reached, the temperature was held for holding times of 0.5 s, 10 s and 30 s. Subsequently, the samples were quenched with a controlled cooling rate of 100 °C/s or with maximum opened gas valve, respectively. Based on this maximum gas quenching, the resulting cooling rate was not similar in each test. In this study, the quenching with maximum opened gas valve is only called “maximum cooling rate”. Hence, regarding the sample nomenclature the abbreviation “max.” stands for “maximum cooling rate”, otherwise 100 °C/s would stand for the slow cooling rate. The parameter combinations were summarized in Table 2.

Using the $l_{0}$ as the initial length, the samples elongation is calculated for each sample according to:

(1) $$\varepsilon =\frac{\Delta l}{l_{0}}$$

where Δl is length change with respect to the initial length. For discussion of the results, also the derivative dε/dT is calculated.

**Figure 2:**
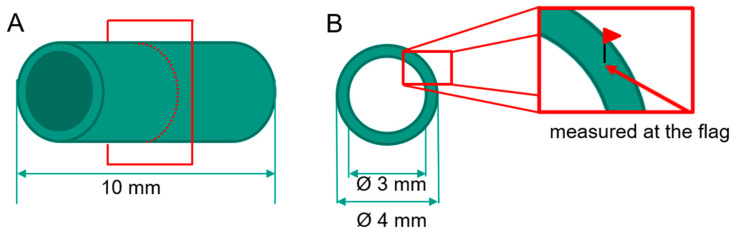
Schematic description of a dilatometer sample, with indication of the cutting plane (**A**) and the position, where the SEM images were taken (**B**).

For metallographic preparation, the samples were cut in the middle position in direction normal to the longitudinal axis. Figure 2 shows a schematic description of a dilatometer sample used in this work indicating the plane of sectioning in Figure 2A and the positions where the SEM images were taken (Figure 2B). One of the two segments was also cut lengthwise. The cut sections were polished using SiC-paper from a grit of P320 to P2500, followed by polishing with diamond suspension with a grain size of 9 μm and lubricant. Before etching with Weck’s reagent, the samples were etch polished with 20 mL H_2_O_2_ and 100 mL OPS (Oxide Polishing Suspension, Struers). On the metallographically prepared samples, the microstructure was investigated using scanning electron microscopy (SEM, LEO EVO 50, Zeiss, Oberkochen, Germany). The martensite phase fraction was determined by means of a point analysis on the micrographs recorded by SEM. At a magnification of 5000, a grid with a total of 345 points was selected. Thus, the grid spacing is about 2.85 μm.

## 3. Results

This section is divided by subheadings. It should provide a concise and precise description of the experimental results, their interpretation as well as the experimental conclusions that can be drawn.

### 3.1. Dilatometer Curves

**Figure 3:**
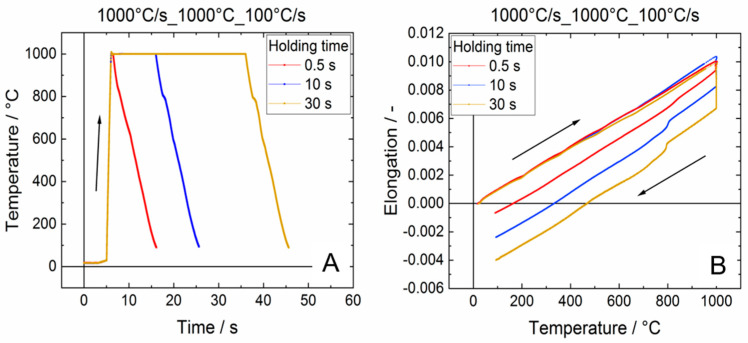
Typical dilatometer results for the alloy Ti6Al4V ELI. Temperature-time-curves (**A**) and corresponding elongation-temperature-distributions (**B**) at the maximum temperature of 1000 °C for the three different holding times 0.5, 10 and 30 s.

In Figure 3, typical dilatometer results are presented. As an example, the temperature–time curves for experiments performed at maximum temperatures of 1000 °C in combination with holding times of 0.5, 10 and 30 s at maximum temperature are presented (Figure 3A). In all cases, a heating rate of 1000 °C/s and a cooling rate of 100 °C/s was applied (nomenclature: 1000 °C/s_1000 °C_100 °C/s). On the right-hand side diagram, the corresponding elongation–temperature distributions are shown.

The distributions were divided into heating, holding and cooling phases and in the following each phase is analyzed separately. According to literature, it was expected that already during heating first transformation of α into β occurs even for temperatures below the β-transus temperature, which is at 975 °C. During holding at high temperature the β content should continuously increase. Pursuant to [19] the α → β transformation is accompanied by a volume decrease, which should cause a measurable effect in the dilatometry experiments. On this basis, it is expected to estimate the β volume shared. However, during heating no clear impact could be detected, because various factors affect the dilatometer measurements at this stage. During heating, the sample continuously expands due to phase-specific thermal strains. The effective expansion of the dilatometer sample would be the sum of the phase-specific expansions, with the respective proportion of the two phases changing with temperature. This effect counteracts the volume decrease due to partial and time-dependent α → β phase transformation. Furthermore, the induction coil does not generate an entirely homogeneous field. In addition, heat conduction occurs towards the glass punches at the edge to the cylindrical sample. Both effects overlap and cause a temperature gradient over the length of the sample. For balancing this transient effect, a sufficiently slow heating and/or a holding step is required. Additionally, at the investigated test temperatures of 950 and 1000 °C, the hot yield strength [20] drops significantly, so creep effects must be taken into account, although the holding forces of the glass punches is rather small (approx. 10 N). This local creep at high temperatures affect the elongation measurement at high temperature during the holding time. In consequence, unfortunately also the holding step is not appropriate to estimate the transformed β-fraction. Hence, for further evaluation of the dilatometer data we focused on the cooling stage only.

**Figure 4:**
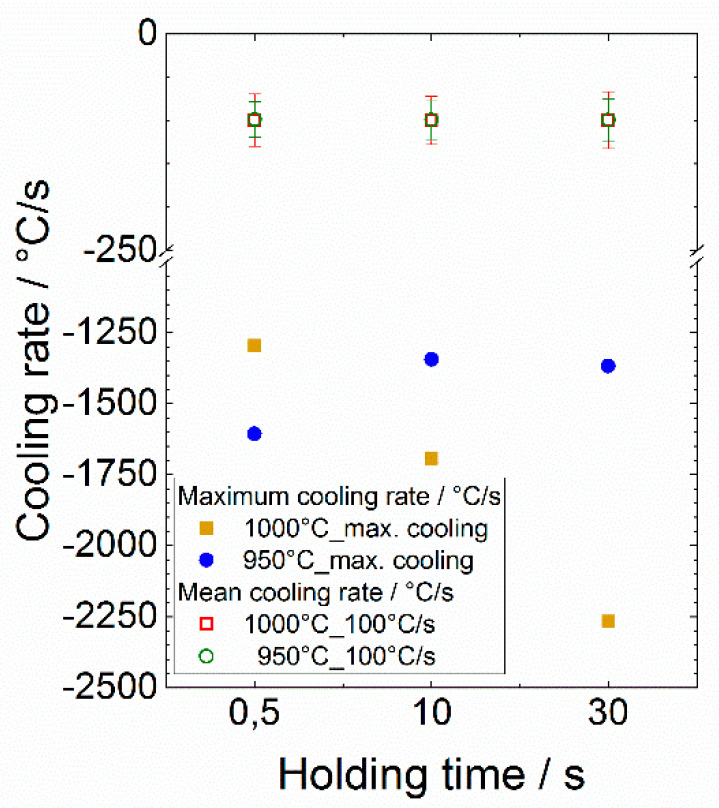
Actual cooling rates applied during the dilatometry for the experiments carried out at the maximum control temperatures of 950 and 1000 °C, respectively.

Here, the maximum cooling rates were realized by opening the helium gas valve entirely, as stated before. Hence, the maximum cooling rates depend on the individual experiment and might differ slightly. In Figure 4, the actual cooling rates are shown as determined by means of the measuring data. The data clearly indicate that for the experiments with a cooling rate of 100 °C/s (controlled process), the set value could be kept well. At maximum cooling, when the gas valve is opened to the maximum, a maximum cooling rate between 1300 and 2250 °C/s is achieved and the cooling rates decrease with time. Due to the continuous change of the cooling rate, the indication of an average cooling rate is neither useful nor meaningful. For the controlled experiments with an average cooling rate of 100 °C/s, an average value was determined in the area of the start of cooling up to 7.5 s after the start.

#### Influence of the Target Temperature on the Martensite Start Temperature

**Figure 5:**
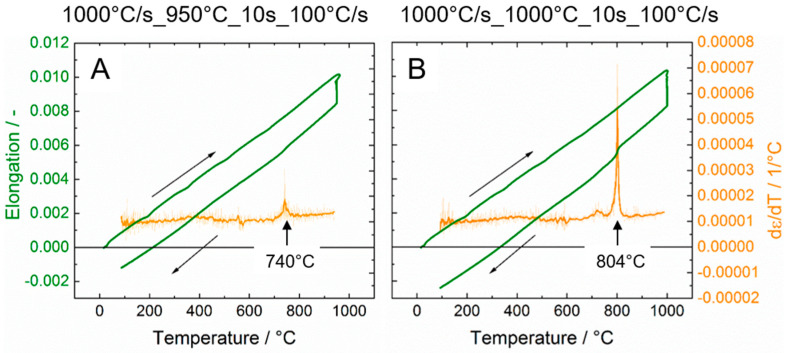
Comparison between elongation-temperature- and dε/dT-distributions for 950 °C (**A**) and 1000 °C (**B**). In both experiments heating was carried out with 1000 °C/s and cooling down at 100 °C/s, respectively, while a holding time of 10 s was applied.

In Figure 5, the elongation (green) and its change over temperature dε/dt (orange, only cooling) vs. process temperature distributions are plotted as an example for the experiments with maximum control temperatures of 950 and 1000 °C and a holding time of 10 s.

For both dilatometer experiments presented in Figure 4, a significant kink in the elongation vs. temperature distribution can be noticed, which clearly indicates the phase transformation β to α’ (martensite). With the help of the dε/dt vs. temperature distribution the start of the transformation (martensite start temperature) can clearly be assigned through a peak that shows up. By this means the martensite start temperature for these individual experiments with 10 s holding time and a cooling rate of 100 °C/s can be determined with sufficient precision to 740 °C for the maximum control temperature of 950 °C and to 804 °C for 1000 °C, respectively.

This evaluation was done for all experiments carried out at 950 and at 1000 °C, respectively, and the result was averaged over same maximum control temperatures. Table 3 summarizes the resulting martensite start temperatures indicating that the martensite start temperature at the higher maximum control temperature of 1000 °C is slightly higher than at 950 °C.

In [21] it is described that a shift of the martensite start temperature can depend on the state of homogenizations of the alloying elements. A significant shift towards higher temperatures and shorter times was observed for a slight increase of oxygen content. Aluminum should have the same effect as oxygen, but the effect is stronger with oxygen.

In the present work the effect of oxygen can be neglected. However, a shift of the martensite start temperature can be caused by an inhomogeneous distribution of the alloying elements, especially aluminum and vanadium. It is known [19] that in an equilibrium state these two elements accumulate unevenly in the two phases. Rapid heating and the associated transformation of the α grains into β leads to segregations in individual β grains. This chemical gradient can only be reduced by means of diffusion during longer holding times, i.e., by homogenization. At the maximum control temperature of 1000 °C the sample was kept longer at higher temperatures than at the maximum control temperature of 950 °C. In consequence, even at short holding times at high temperatures the distribution of aluminum or vanadium in the sample at 1000 °C might be more homogenized than in the experiment at 950 °C. EDS analysis indicated, that for the sample heat treated at 1000 °C for 0.5 s holding time followed by maximum quenching the elemental distribution is inhomogeneous and showed a bimodal distribution. Locally, elemental distributions with 4.8 wt.% Al and 7.3 wt.% V were observed, which clearly differ to the nominal distribution. However, based on the SEM images even at high magnifications no clear assignments could done to the underlying microstructure. However, after holding time for 30 s at 1000 °C followed by maximum cooling a homogeneous elemental distribution was observed according to the nominal alloy composition and XRD phase analysis revealed a slightly lower β-content with only about 2.1 ± 0.4 vol.% in comparison with the initial state.

### 3.2. Metallography

**Figure 6:**
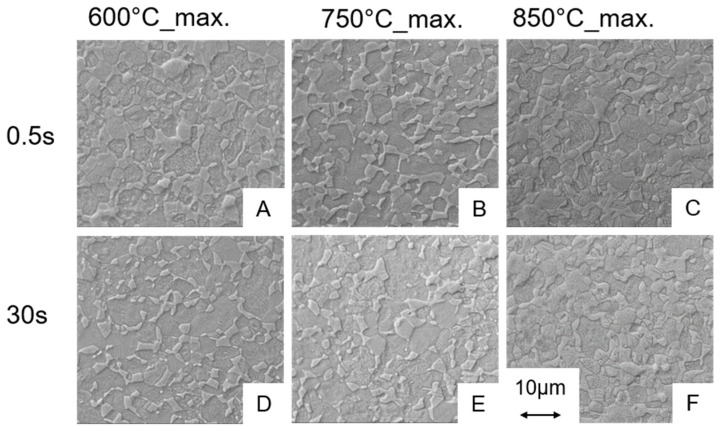
SEM images of the microstructures for the experiments at 600, 750 and 850 °C for holding times of 0.5 (**A**–**C**) and 30 s (**D**–**F**) in combination with maximum cooling. The heating rate was 1000 °C/s.

**Figure 7:**
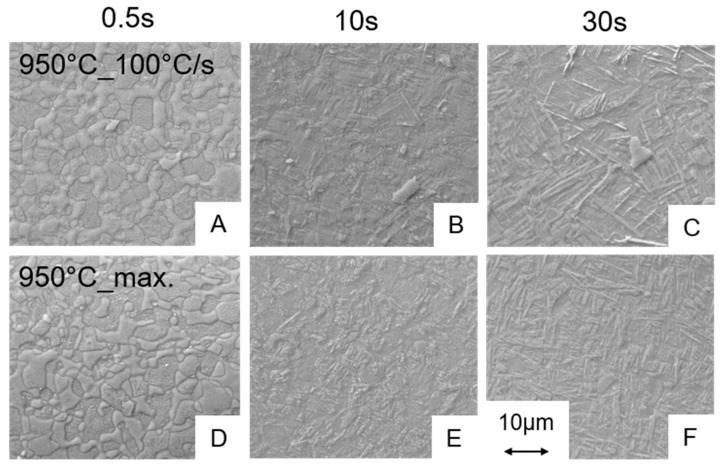
SEM images of the microstructures for the experiments at 950 °C for holding times of 0.5, 10 and 30 s in combination with controlled cooling with 100 °C/s (**A**–**C**) and with the maximum cooling rate (**D**–**F**). The heating rate was 1000 °C/s.

For the assessment of the microstructures, SEM images are recorded at a magnification of 5000×. In Figure 6, the micrographs for the experiments carried out at the maximum control temperature of 600, 750 and 850 °C are shown only for the maximum cooling rates for the holding times of 0.5 s (Figure 6A–C) and 30 s (Figure 6D–F), respectively. The SEM investigations for samples from the experiments carried out at maximum control temperatures of 600, 750 and 850 °C indicated no significant changes of the microstructure compared to the as received state (Figure 1), i.e., for all variants using these maximum control temperatures the microstructures all fulfil the requirements from the above mentioned standards for medical applications. That means that even for processing at the maximum control temperature of 850 °C (Figure 6C,F) and a maximum cooling rate, the resulting microstructure is in good agreement with the one of the initial state, independent of the holding time. However, after a holding time of 30 s the microstructure resulting from the experiment at 850 °C with maximum cooling shows a slight change at the grain boundaries compared to the as-received state, i.e., the grain boundaries appear more roundish (Figure 6F). However, the microstructure still fulfils the requirements for medical applications. The same observations were made for the microstructures from the experiments 950 °C_0.5 s_100 °C/s and 950 °C_0.5 s_max (Figure 7A,D).

**Figure 8:**
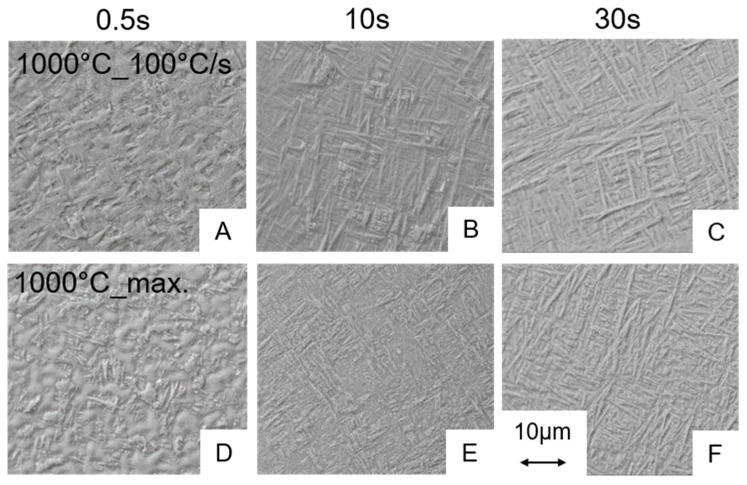
SEM images of the microstructures for the experiments at 1000 °C for holding times of 0.5, 10 and 30 s in combination with controlled cooling with 100 °C/s (**A**–**C**) and with the maximum cooling rate (**D**–**F**). The heating rate was 1000 °C/s.

Conversely, this means that during the short-term heat treatment procedures the holding time is most important for the amount of β that develops in the microstructure being available for martensite formation during cooling down. Since almost no microstructural changes up to maximum control temperature of 850 °C occurred, we assume that no significant amount of β-phase was formed in case of the thin walled hollow samples even at the longest holding time of 30 s. Martensite start temperature for 950 °C as maximum control temperature was about 756 °C (see Table 3). Hence, since no martensite was observed in the microstructure of the sample short-term heat treated at 850 °C, this observation supports the conclusion that no significant phase fraction of β was formed in the 850 °C process variants. Regarding the parameter window studied in this project, the micrographs and the dilatometry results indicate that a maximum control temperature of 950 °C in combination with a holding time of 10 s or more is necessary to form martensite for the cooling rates of ≥100 °C/s. Experiments carried out at 1000 °C show also a martensitic transformation independently of the holding time and the cooling rate. Only the amount of martensite formed differs (Figure 8). For maximum control temperatures of up to 950 °C in combination with a short holding time of 0.5 s, no martensitic phase fractions could be detected by means of SEM. Taking into account the microstructures listed and permitted in the standard [16] for medical products, all heat treatments without martensite formation are acceptable for medical applications.

#### 3.2.1. Influence of the Heating Rate on the Microstructure

**Figure 9:**
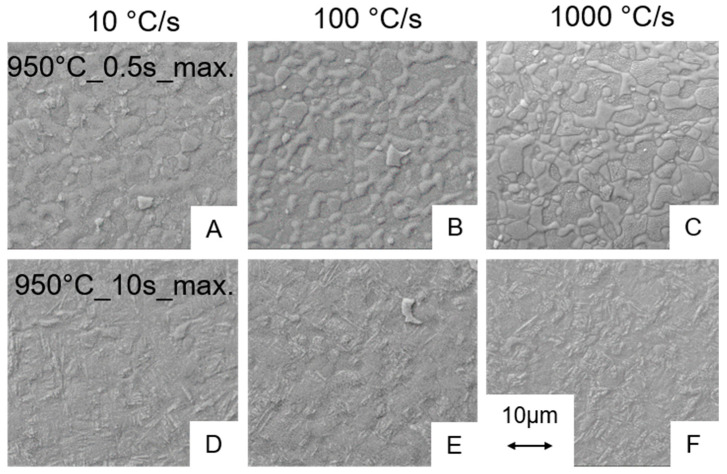
SEM images of the microstructures for the experiments at 950 °C for heating rates of 10 °C/s, 100 and 1000 °C/s in combination with holding times of 0.5 s (**A**–**C**) and 10 s (**D**–**F**), respectively.

The statements made so far, all hold for heating rates of 1000 °C/s. However, it is still unknown if slower heating rates affect the microstructure evolution, since at slower heating rates more time for homogenization exist. For this reason, heating rates of 100 °C/s and even at 10 °C/s were considered for the maximum control temperature of 950 °C. The resulting microstructures are presented in Figure 9 for holding times of 0.5 s (Figure 9A–C) and for 10 s (Figure 9D–F), respectively.

However, the micrographs indicate that the heating rate has no significant effect on the resulting microstructure. It can be assumed that the longer time period available for homogenization does not affect the microstructure evolution. For the holding time of 0.5 s, no further impact on the microstructure formation can be noticed. At a holding time of 10 s, a small martensite fraction can be seen for all three heating rates observed and obviously the heating rate has a neglectable influence on the phase fraction, which is almost the same for all three process variants. That means that even a relatively slow heating rate of 10 °C/s in combination with a holding time of 0.5 s is far from an equilibrium treatment as no significant β-phase fraction forms.

#### 3.2.2. Influence of the Holding Time and Cooling Rate on the Martensite Content

**Figure 10:**
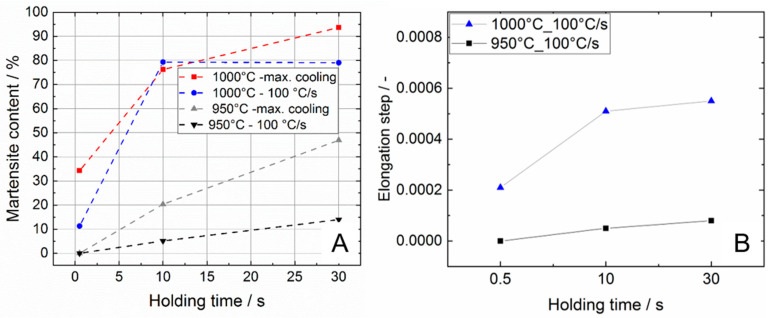
Comparison between martensite vs. holding time distribution (**A**) and elongation step vs. holding time distribution (**B**) for maximum control temperatures of 950 and 1000 °C.

The micrographs presented before reveal that the martensite phase fraction strongly depend on the holding time of the short-term heat treatments for maximum control temperatures of ≥950 °C. Regarding the dilatometer curves the martensite formation results in a distinct step in the elongation vs. temperature distribution during cooling down. It was examined if the step height correlates with the martensite phase fraction observed by means of microscopy and image analysis. For this purpose, the course of the elongation vs. temperature distribution before and after the step was fitted linearly and the elongation step was determined. In Figure 10, the metallographically determined martensite phase fraction (Figure 10A) is compared with this step height (Figure 10B). A clear correlation can be seen between the two diagrams. For the experiments with a cooling rate of 100 °C/s and a test temperature of 950 °C, an almost linear increase of the step height with an increase of the holding time can be observed. This observation is closely consistent with the metallographic findings. In contrast, no linearity can be detected for the test temperature of 1000 °C. The step height increases sharply between the holding time of 0.5 and 10 s, respectively. Only a slight further increase can only be observed between holding times of 10 and 30 s. This behavior also clearly correlates with the metallographic findings. The data reveal that for the combination of 1000 °C test temperature with a holding time of 30 s and 100 °C/s cooling rate a martensite phase fraction of about 78% develops. However, at maximum cooling an almost completely martensitic structure occurred. Hence, the limiting factor for martensite formation is surely the cooling rate. As a consequence, the cooling rate of 100 °C/s is obviously not high enough for a complete martensitic transformation of the structure. The image analysis further indicated that a holding time of 30 s at 1000 °C, which is slightly above the β-transus temperature of the alloy is enough to transform the initial α-β-structure completely to β. For a temperature slightly below β-transus with identical heating and cooling rate and also 30 s holding time results in a martensite content of approximately 47%. For experiments at maximum control temperatures of ≤850 °C no step in the elongation vs. temperature dilatometry curve can be observed that is in good agreement with the microstructural analysis. Here, no martensite could be found by means of microscopy. It is likely that both the β-phase fraction and the necessary supercooling are insufficient for a noticeable martensite formation, although the maximum control temperature should be above the (short-term heat treatment) martensite start temperature. The findings of microscopy analysis correspond well with dilatometry measurements; hence, the elongation vs. temperature distribution in combination with the dε/dT-curve is appropriate to assess martensitic transformation of the alloy Ti6Al4V ELI.

**Figure 11:**
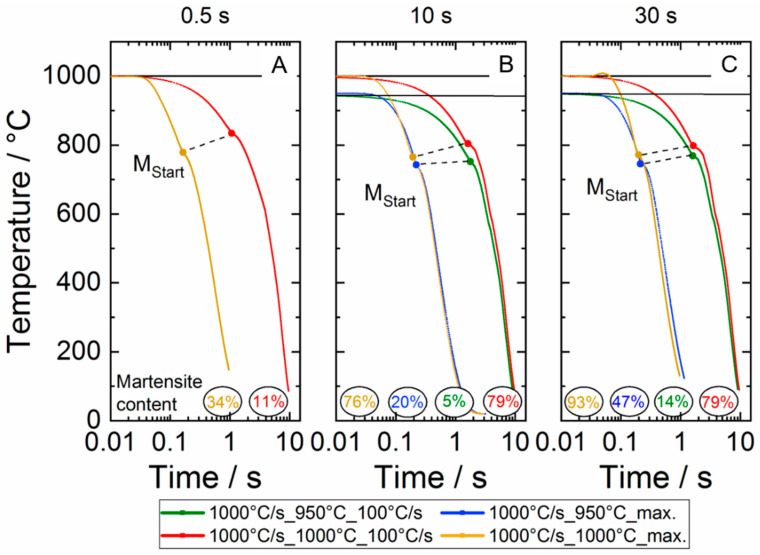
Time–temperature transformation relationship of the alloy Ti6Al4V ELI during continuous cooling down using a controlled cooling rate of 100 °C/s and maximum cooling (cooling rates above 1000 °C/s—see Figure 4) for holding times of 0.5 s (**A**), 10 s (**B**) and 30 s (**C**), respectively. The buckling points clearly indicate the martensite start temperatures.

Finally, the results of the short-term heat treatment of Ti6Al4V ELI are graphically summarized in a plot characterizing the time–temperature transformation behavior for the herein considered process variants. Figure 11 shows the cooling curves from the dilatometer experiments for the experiments carried out at a maximum control temperature of 950 and 1000 °C for the holding times 0.5 s (Figure 11A), 10 s (Figure 11B) and 30 s (Figure 11C), respectively. The martensite start temperatures, as well as the resulting martensite phase fractions, are indicated. Hence, the diagrams resemble continuous time–temperature transformation diagrams. The presented data indicate that the martensite start temperature slightly increases as the cooling rate decreases and tends to fall slightly as the holding time increases. For maximum control temperatures ≤850 °C, no martensite formation was observed independent on the holding time and cooling rate applied. Test temperatures below 950 °C reveal no abnormalities in the course.

## 4. Conclusions

Dilatometry tests were carried out for thin walled hollow samples to characterize the short-term heat treatment behavior of the alloy Ti6Al4V ELI for maximum control temperatures between 600 and 1000 °C using holding time at maximum temperatures of 0.5, 10 and 30 s, respectively. Very fast cooling was carried out by controlled cooling with a rate of 100 °C/s and maximum cooling which results in cooling rates >1000 °C/s. The materials’ behavior was characterized by means of microstructure analysis using SEM and assessment of the elongation vs. temperature distributions determined by dilatometry. From the investigations, it can be concluded for the material state that was initially heat treated at 900 °C for 10 min under vacuum followed by oil quenching and finally annealed at 500 °C for 4 h followed by a slow furnace cooling, that

- The alloy Ti6Al4V ELI can be heat treated up to 850 °C using a heating rate of 1000 °C/s and a holding period at maximum temperature of 30 s without significantly changing the initial α-β-microstructure, which is acceptable for medical applications according to [16,17];
- Short-term heat treatment with a maximum temperature to 950 °C, which is slightly below the β-transus temperature, and very short holding times of 0.5 s, also cause no significant phase transformations. However, heat treatment with temperatures ≥950 °C and comparable heating and cooling rates should not take longer than 0.5 s; otherwise, the resulting microstructure will no longer fulfil the requirements for medical applications;
- Heating rates between 10 and 1000 °C/s showed no significant influence on the resulting microstructure during short-term heat treatments;
- The cooling rate of 100 °C/s is too low to trigger a complete transformation of the β phase into martensite for maximum temperatures of 950 and 1000 °C, respectively;
- The cooling rates obviously do not affect the martensite start temperature in contrast to the maximum control temperature. Here, an increase in the maximum temperature results in a slight increase in the martensite start temperature, which is due to the degree of chemical homogenization;
- The analysis of the elongation kink in the dilatometer curve, which appears when the martensite transformation sets in, can be used to assess the martensite phase fraction that has been transformed during quenching from high maximum temperatures. The results are in good agreement with metallographic analysis.

## Figures and Tables

**Table 1:** Materials properties of Ti6Al4V ELI (measured data).

Young’s Modulus	Yield Strength	Tensile Strength	Vickers Hardness
106 ± 0.8 GPa	910 ± 5 MPa	967 ± 5 MPa	321 ± 8 HV_0.1_

**Table 2:** Parameter settings realized in the dilatometer experiments.

Target Temperatures	Heating Rates	Holding Times	Cooling Rates
600, 750, 850, 950, 1000 °C	10, 100, 1000 °C/s	0.5, 10, 30 s	100 °C/s, max. cooling

**Table 3:** Averaged martensite start temperature for maximum control temperatures of 950 and 1000 °C for experiments carried out using a holding time of 10 s and a cooling rate of 100 °C/s.

Maximum Control Temperature	Martensite Start Temperature
950 °C	756 ± 17 °C
1000 °C	805 ± 13 °C
